# Changes of Tumor Markers in Patients with Breast Cancer during Postoperative Adjuvant Chemotherapy

**DOI:** 10.1155/2022/7739777

**Published:** 2022-05-19

**Authors:** Yan Zhang, Jing Zhao, Yajun Wang, Wei Cai, Xiaoli Zhang, Kaifu Li, Wenqing Liu, Ye Zhao, Hua Kang

**Affiliations:** Department of General Surgery, Xuanwu Hospital, Capital Medical University, China

## Abstract

**Objective:**

Serum tumor marker (STM) elevation can detect metastasis earlier than imaging diagnosis and, although not recommended by guidelines, is still widely used in clinical practice for postoperative follow-up of breast cancer patients. The purpose of this study was to investigate the change rules of CEA and CA153 in patients with HER2-negative breast cancer during postoperative adjuvant chemotherapy and their influencing factors.

**Materials and Methods:**

The medical records of patients with HER2-negative early breast cancer who visited Xuanwu Hospital from September 2018 to June 2021 were retrospectively analyzed. Demographic characteristics and baseline data of CEA and CA153 at initial diagnosis were collected. Data of CEA, CA153, biochemistry (including ALT, AST, rGT, triglycerides, cholesterol, and blood glucose) and blood routine (including white blood cells, neutrophils, monocytes, lymphocytes, and platelets) were also collected before chemotherapy, at the end of chemotherapy and more than 3 months after the end of chemotherapy. LY/MONO, NEUT/LY, PLT/LY, and systemic immune inflammation index (SII) were calculated and statistically analyzed using SPSSAU software.

**Results:**

A total of 90 patients were enrolled, all of whom were female, with a mean age of 55.11 ± 10.60 y. The value of CEA at initial diagnosis was 2.10 ± 1.11 ng/mL, and high expression was mostly correlated with past history of chronic diseases and tumor lymph node metastasis; the value of CA153 was 11.80 ± 6.60 U/mL, and high expression was correlated with high SII at initial diagnosis. Surgery did not affect the values of serum CEA and CA153. At the end of chemotherapy, CEA and CA153 were 2.68 ± 1.34 ng/mL and 18.51 ± 8.50 U/mL, respectively, which were significantly increased compared with those before chemotherapy, and were linearly correlated with the values before chemotherapy. They decreased (CEA 2.45 ± 1.19 ng/mL, CA153 10.87 ± 5.96 U/mL) again three months after the end of chemotherapy, manifested as “spiking” phenomenon, which was associated with lymph node metastasis at diagnosis, body metabolic disorders, and chronic inflammatory status.

**Conclusion:**

CEA and CA153 were increased presenting as “spiking” phenomena in patients with early HER2-negative breast cancer during postoperative adjuvant chemotherapy, and the peak of increase was linearly correlated with the indicators before chemotherapy. Clinical attention should be paid to this change to avoid excessive diagnosis and treatment leading to medical resource consumption.

## 1. Introduction

Latest global cancer data in 2020 released by the International Agency for Research on Cancer (IARC) of the World Health Organization showed that there were about 19.3 million new cancer patients worldwide in 2020, of which breast cancer accounted for 11.7%, becoming the cancer with the most newly diagnosed people in the world; the number of deaths for breast cancer was 680,000, and the number of deaths ranked fourth among all cancers [1]. How to develop a reasonable follow-up strategy for postoperative breast cancer patients and timely detect recurrence and metastasis is a concern for both doctors and patients. Unfortunately, there is a lack of effective clinical markers to help detect patients' recurrences and metastases in early stage. In recent years, some studies [2–4] have shown that the detection of ctDNA, long noncoding RNA, and modulated microRNAs in patients' body fluids can be detected by liquid biopsy for the recurrences and metastases of patients in early stage and is a promising prognostic marker. However, these marker detection with these methods are expensive, the detection methods are not standardized, they are still in research stage, and they have not been applied in clinical practice as a method to monitor tumor metastasis.

Serum tumor marker (STM) has become a powerful tool for patient follow-up in many tumor species due to its advantages of easy clinical availability, dynamic monitoring, minimal invasiveness, and low cost of testing [5, 6]. Using the follow-up modality of dynamic monitoring of STM, the elevation of STM can occur 4–6 months before imaging diagnosis of tumor metastasis, which facilitates the detection of early metastasis without clinical symptoms [7–9]. However, due to the lack of survival benefit data, neither the European Society for Medical Oncology (ESMO) nor the American Society of Clinical Oncology (ASCO) recommend dynamic monitoring of changes in STM as part of the postoperative follow-up of early breast cancer [10, 11]. However, previous studies in which close postoperative follow-up could not improve the survival of patients were carried out earlier [12, 13]; in recent years, the progress of drugs, especially the advent of various targeted drugs, has significantly prolonged the survival of patients with metastasis. Therefore, the results of previous studies may not be applicable to current clinical practice. For the above reasons, many centers are still applying STM as a monitoring index for postoperative follow-up of breast cancer [14–16]. Of course, the strategy of dynamic monitoring of STM also has some defects: because STM is affected by a variety of nontumor factors, the increase of STM may be a false positive result in some cases [17–19], and such a false positive result will lead to patient anxiety and consume unnecessary medical resources; therefore, understanding the factors leading to false positive STM during postoperative follow-up of breast cancer is important for the follow-up of breast cancer patients.

Postoperative adjuvant chemotherapy is an important treatment for breast cancer, adjuvant chemotherapy reduced breast cancer mortality by, on average, about one-third [20]. Postoperative adjuvant chemotherapy was listed as the standard treatment for breast cancer by the NCCN and ESMO guidelines. The spike phenomenon of STM can occur in patients with advanced breast cancer after chemotherapy or endocrine therapy [21], and the spike phenomenon is not related to tumor progression. Whether the adjuvant chemotherapy phase will also produce a similar phenomenon is not clear to us due to the lack of relevant clinical studies. Therefore, the purpose of this study is to investigate the changes of CEA and CA153 in patients with early breast cancer during postoperative adjuvant chemotherapy, providing more reference for clinical breast cancer follow-up.

## 2. Materials and Methods

### 2.1. Patients

The medical records of patients with early breast cancer who visited Xuanwu Hospital from January, 2019 to September, 2021 were retrospectively analyzed. Inclusion criteria are as follows: In cases of pathologically diagnosed early HER2-negative breast cancer, surgery was performed first after diagnosis, then followed by postoperative adjuvant chemotherapy, and then followed by other treatments, such as radiotherapy or endocrine therapy, and with complete clinical data. Exclusion criteria were as follows: Incomplete clinical data, patients receiving neoadjuvant therapy, patients with HER2-positive disease requiring targeted therapy, patients with recurrence or metastasis within 6 months after the end of chemotherapy, patients with a history of other malignancies, and patients who have previously received chemoradiotherapy.

### 2.2. Review of Clinical Data

The HIS case system of Xuanwu Hospital was reviewed, and the clinical and laboratory data of the patients were collected. Clinical data included patient's age, menstrual status, height, weight, comorbidities (history of chronic diseases such as hypertension, hyperlipidemia, diabetes, coronary heart disease, and chronic lung disease), patient's surgical approach, tumor size, nodal status, immunohistochemical markers (estrogen receptor, progesterone receptor, HER2), and postoperative chemotherapy regimen. Laboratory data were recorded simultaneously, including CEA, CA153 at initial diagnosis, biochemical indicators (including alanine aminotransferase (ALT), aspartate aminotransferase (AST), r-glutamyltransferase (rGT), fasting blood glucose, triglyceride and cholesterol), blood routine (including WBC, NEUT, MONO, LY and PLT); CEA, CA153 and biochemical indicators before chemotherapy (same as before); CEA, CA153 and biochemical indicators at the end of chemotherapy (same as before); and expression of CEA and CA153 of patient after 3 months since the end of chemotherapy. The study design and method were approved by the Ethics Committee of Xuanwu hospital.

### 2.3. The Calculation Formula of Relevant Indicators

The calculation formula of relevant indicators is as follows:

BMI = weight (kg)/height (m)^2^, 

Magnitude of change = (end − of − chemotherapy value − prechemotherapy value)/prechemotherapy value, 

PLT/LY = platelet count/lymphocyte count, 

NEUT/LY = neutrophil count/lymphocyte count, 

LY/MONO = lymphocyte count/monocyte count, 

Systemic immune inflammation index (SII) = (neutrophil count × platelet count)/lymphocyte count.

### 2.4. Statistical Analyses

The continuous variables were expressed as mean ± standard deviation; categorical variables were expressed as numbers. The effects of various factors on CEA and CA153 were analyzed by one-way ANOVA and multivariate analysis of variance, the trends of CEA and CA153 at different time points were analyzed by variance analysis, and the relationship between CEA and CA153 before and after chemotherapy was analyzed by correlation analysis and calculate the regression equation. All of the reported *P* values were two-sided, and *P* values < 0.05 were considered statistically significant. Statistical analyses were carried out by SPSSAU (Statistical Product and Service Software Automatically, https://spssau.com/) (Beijing Qingsi Technology Ltd.).

## 3. Results

### 3.1. Demographic Characteristics

A total of 90 patients were enrolled, all of whom were female, and the clinicopathologic features were summarized in Table 1. The median age of the patients was 55.11 ± 10.60 y (range 28-74 y). Thirty-nine (43.3%) patients had comorbidities, including hypertension, hyperlipidemia, diabetes, coronary heart disease, and chronic lung disease, and 69 (76.7%) patients had hormone receptor (estrogen receptor and progesterone receptor) positive. The normal range was 0.01-5 ng/mL for CEA and 0.01-25 U/mL for CA153. Other clinicopathological features are detailed in Table 1.

### 3.2. Analysis of the Influencing Factors of CEA and CA153 at Initial Diagnosis

The values of CEA and CA153 at initial diagnosis were 2.10 ± 1.11 ng/mL and 11.80 ± 6.60 U/mL, respectively. We analyzed the relationship between the expression of CEA and CA153 vs. clinicopathology and laboratory indicators in patients at initial diagnosis. Univariate analysis revealed that the high expression of CEA was associated with older age, menopause, complications, tumor lymph node metastasis, and higher liver function in early breast cancer patients without metastasis (see Table 2), and multivariate analysis showed that the high expression of CEA was associated with complications and tumor lymph node metastasis, indicating that the expression of CEA was associated with metabolic disorders and tumor stage. The high expression of CA153 was correlated with the high systemic immune-inflammatory index SII in univariate and multivariate analysis (see Table 3), indicating that the expression of CA153 is related to the chronic inflammatory state of the body.

### 3.3. Effect of Adjuvant Chemotherapy on CEA and CA153

**Figure 1:**
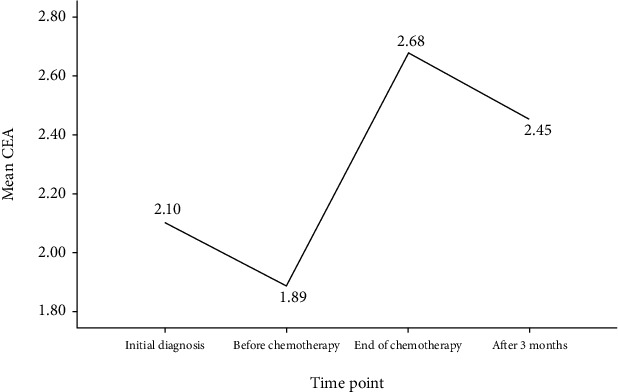
Changes of CEA during postoperative adjuvant chemotherapy: CEA value (ng/mL) was the highest at the end of chemotherapy, which was significantly higher than those of the initial diagnosis and before chemotherapy, and the difference was statistically significant, *P* < 0.01; the CEA value decreased 3 months after the end of chemotherapy, but it was not statistically significant.

**Figure 2:**
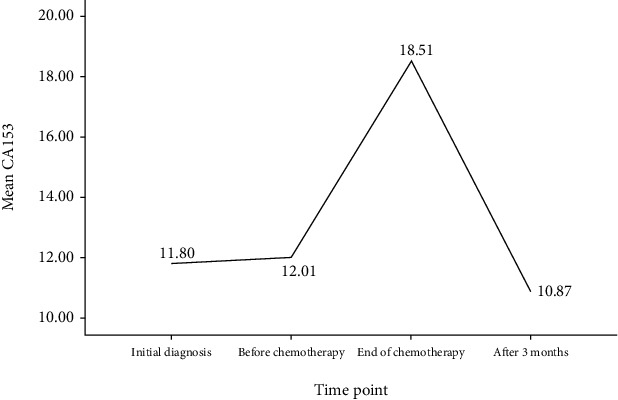
Changes of CA153 in postoperative adjuvant chemotherapy stage: CA153 value (U/mL) was the highest at the end of chemotherapy, which was significantly higher than those of the initial diagnosis and before chemotherapy, and the difference had statistical significance, *P* < 0.01; CA153 value showed a decrease 3 months after the end of chemotherapy, which was significantly different from that of the end of chemotherapy, *P* < 0.01.

All patients received surgical treatment first, chemotherapy was started 3–4 weeks after surgery, and chemotherapy was followed by radiotherapy and/or endocrine therapy according to the medical condition. See Table 4 for detailed data of CEA and CA153 of patients at different time points. The results of multiple comparisons afterwards showed that CEA and CA153 increased significantly after chemotherapy compared with those at initial diagnosis and before chemotherapy, and all *P* < 0.01. Three months after the end of chemotherapy, it showed a decrease, presenting a typical spike phenomenon, as shown in Figures 1 and 2 (the number of cases with tumor markers detected 3 months after the end of chemotherapy: *n* = 83, because the patients were in other cities and did not undergo tumor marker detection).

**Figure 3:**
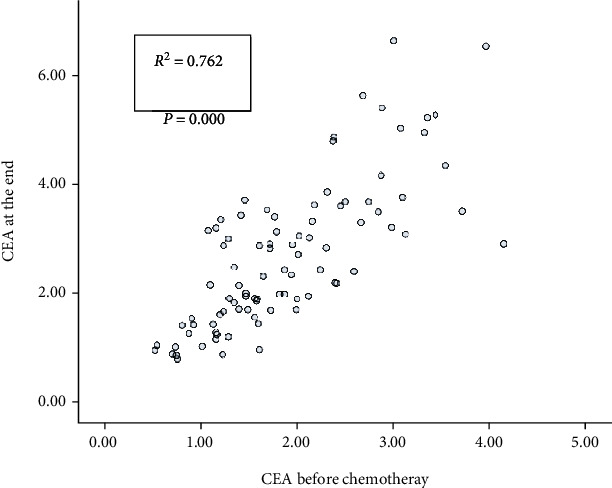
Correlation in CEA values between prechemotherapy and at the end of chemotherapy (scatter diagram).

**Figure 4:**
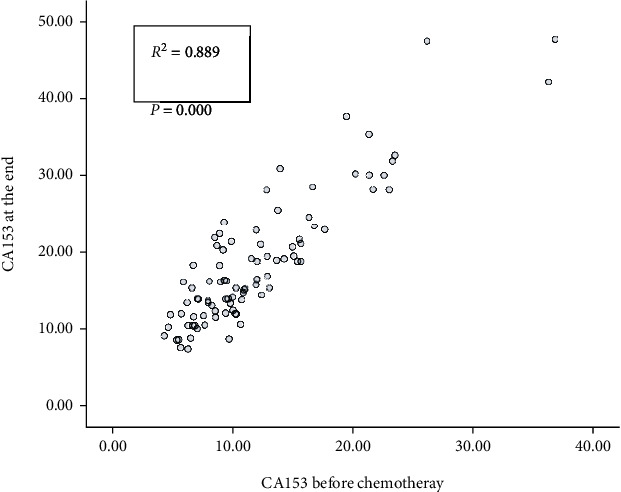
Correlation in CA153 values between prechemotherapy and at the end of chemotherapy (scatter diagram).

Subsequently, we plotted the scatter diagrams of CEA and CA153 in patients before chemotherapy and at the end of chemotherapy (Figures 3 and 4) and found that the value of CEA at the end of chemotherapy was linearly correlated with the value before chemotherapy, with a Pearson correlation coefficient of 0.762, *P* < 0.001, and the regression equation was CEA at the end = 0.340 + 1.238^∗^ CEA before chemotherapy, and the model passed the *F*-test, *F* = 121.881, *P* < 0.001. The value of CA153 at the end of chemotherapy was linearly correlated with the value before chemotherapy, with a Pearson correlation coefficient of 0.889, *P* < 0.001, and the regression equation was CA153 at the end = 3.923 + 1.215^∗^ CA153 before chemotherapy, and the model passed the *F*-test, *F* = 330.056, *P* < 0.001.

### 3.4. Analysis of Influencing Factors of CEA and CA153 Increase after Chemotherapy

In view of the phenomenon of CEA and CA153 increase during adjuvant chemotherapy, we further explored the related influencing factors. Our study showed that the large change magnitude of CEA before and after chemotherapy was associated with multiple lymph node metastasis, negative hormone receptor, low NEUT/LY at initial diagnosis, and increased rGT in patients, and multivariate analysis showed that these four indicators were all factors influencing the CEA increase during chemotherapy. The CA153 change magnitude during chemotherapy was correlated with the patient's age, comorbidities, lymph node metastasis status, initial WBC and NEUT values, and multivariate analysis showed that only multiple lymph node metastasis was a factor influencing the CA153 change magnitude (see Tables 5 and 6).

## 4. Discussion

CEA is a glycoprotein and consists of 45 percent protein and 55 percent carbohydrate, which was involved in cell adhesion [22]. CA15-3 is carbohydrate-containing protein antigen of the transmembrane glycoprotein MUC-1, which appears to inhibit tumor cell lysis and reduce cell-cell interactions [23]. These two tumor markers are widely adopted in breast cancer management. In the postoperative follow-up stage of early breast cancer, ESMO and ASCO guidelines do not recommend routine monitoring of STM due to lack of survival benefit, but it is still widely used in clinical practice [14–16]. The reason is that previous clinical trials have been the result of limited drug treatment in the past. At present, antitumor drugs have made great progress, so that patients with breast cancer can receive multiple lines of effective treatment. In such a situation, patients with early detection of recurrent metastases may have the opportunity to receive more effective treatment, thus to improve survival. Because STM detection is simple and easy to perform, with low cost, and medical insurance bears expenses, and it can detect metastasis in early stage; therefore, our center currently recommends STM detection for the follow-up of patients with early breast cancer. In this study, only the change rule of STM in HER2-negative breast cancer was investigated, because patients with HER2-positive breast cancer will not only receive chemotherapy but also receive targeted therapy, with many influencing factors, so it was not included in this study population.

The results of this study showed that the high expression of CEA at initial diagnosis was associated with lymph node metastasis and chronic diseases such as diabetes, hypertension, and hyperlipidemia, and the high expression of CA153 was associated with high SII. In the postoperative adjuvant chemotherapy stage, both CEA and CA153 showed “spiking” phenomenon, which was low before chemotherapy, and increased after chemotherapy, and decreased again 3 months after the end of chemotherapy. There was a linear correlation between the STM after chemotherapy and the value of STM before chemotherapy. To our knowledge, this is the first retrospective study to analyze the change rules of tumor markers during the adjuvant chemotherapy phase. Since none of our cases had recurrence or metastasis within 6 months after the end of chemotherapy, therefore, “spiking” phenomenon was not associated with tumor progression. The changes suggest that the spiking phenomenon of STM should be considered when we dynamically observe the changes of tumor markers during the adjuvant chemotherapy stage, especially in patients with high baseline STM, so as to avoid excessive examination and treatment.

For STM “spiking” phenomenon during the adjuvant chemotherapy phase, we also explored some influencing factors. Previous studies have shown that changes in tumor markers are not only associated with tumor lesions but also with many benign lesions and the patient's own physical condition. In patients with poorly controlled diabetes, an increase in CEA can be observed [24, 25]. The expression of CEA correlates with age [26], smoking [27], glucose and lipid metabolism disorders [28, 29], and the chronic inflammatory state of the body [30]. However, CA153 was also significantly increased in diseases such as renal impairment in type 2 diabetes [31], interstitial lung disease [32], idiopathic inflammatory myositis [33], sarcoidosis [34], and SARS-CoV-2 infection [35]. Adjuvant chemotherapy can aggravate blood glucose and blood lipid metabolic disorders while killing potential tumors [36, 37], leaving the body in a state of chronic inflammation. Our analysis showed that “spiking” phenomenon was not only correlated with lymph node metastasis and tumor hormone receptor status but also correlated with the change of rGT and the ratio of NEUT/LY. The increase of rGT was positively correlated with metabolic syndrome [38], indicating that tumor factors, body metabolic factors and chronic inflammatory status will all affect the expression of STM.

Some limitations of our study design should be noted. Firstly, due to the limited number of cases, this study did not perform stratified analysis based on the clinical pathological features of patients; meanwhile, due to the lack of long-term follow-up data of patients in this study, the difference between the increase of tumor markers due to the change of internal environment of the body and the increase of tumor markers due to early tumor recurrences cannot be distinguished. The study was a retrospective study, the number of cases was also small, and the conclusion needs to be further confirmed by the study with greater sample size.

In conclusion, in the postoperative adjuvant chemotherapy stage of patients with early HER2-negative breast cancer, CEA and CA153 showed “spiking” phenomenon, and the increased peak value was linearly correlated with the STM value before chemotherapy. The phenomenon was correlated with the chronic inflammatory state of the body and glucose and lipid metabolic disorders during chemotherapy, suggesting that clinical attention should be paid to this change to avoid excessive diagnosis and treatment.

## Figures and Tables

**Table 1:** General characteristics of the study population (*n* = 90).

Characteristics	*N* (*n* = 90)	%
Age (years)		
≤57	45	50.0
> 57	45	50.0
Menstrual status		
No	56	62.2
Yes	34	37.8
BMI		
≤24	40	44.4
> 24	50	55.6
Comorbidities		
No	51	56.7
Yes	39	43.3
Tumor size		
≤ 2 cm	46	51.1
> 2 cm	44	48.9
Nodal status		
0	48	53.3
1-3	30	33.3
≥ 4	12	13.3
HR status		
Positive	69	76.7
Negative	21	23.3
Adjuvant chemotherapy		
Taxane	48	53.3
Anthracycline and taxane	42	46.7
Surgery in breast		
BCS	35	38.9
Mastectomy	55	61.1

HR: hormone receptor; BCS = breast-conserving surgery.

**Table 2:** Correlation between CEA levels and clinicopathological factors at initial diagnosis.

Factor	CEA value ng/mL (mean ± SD)	Univariate analysis		Multivariate analysis	
		*F*	*P*	*F*	*P*
Age					
≤57	1.77 ± 1.03	8.872	0.004	1.344	0.250
> 57	2.44 ± 1.10				
Menstruation					
Yes	1.73 ± 1.05	6.702	0.011	0.263	0.609
No	2.33 ± 1.09				
Comorbidities					
No	1.78 ± 0.88	11.348	0.001	4.819	0.031
Yes	2.53 ± 1.24				
BMI					
≤24	1.98 ± 1.09	0.865	0.355		
>24	2.20 ± 1.13				
Tumor size					
≤2 cm	1.94 ± 1.04	1.935	0.168		
>2 cm	2.27 ± 1.17				
Nodal status					
0	1.83 ± 0.88	3.597	0.032	3.260	0.043
1-3	2.49 ± 1.36				
≥4	2.22 ± 1.03				
HR status					
Positive	2.21 ± 1.15	2.787	0.099		
Negative	1.75 ± 0.90				
ALT at initial diagnosis					
≤15	1.88 ± 1.00	3.806	0.054		
>15	2.33 ± 1.18				
AST at initial diagnosis					
≤20	1.86 ± 1.00	5.283	0.024	1.781	0.186
>20	2.38 ± 1.18				
rGT at initial diagnosis					
≤16	2.12 ± 1.09	0.024	0.876		
>16	2.09 ± 1.14				
Triglycerides at initial diagnosis					
≤1.09	2.01 ± 1.01	0.608	0.438		
>1.09	2.19 ± 1.20				
Cholesterol at initial diagnosis					
≤4.62	2.04 ± 1.13	0.264	0.609		
>4.62	2.16 ± 1.10				
Fasting blood glucose at initial diagnosis					
≤4.88	1.93 ± 0.98	2.228	0.139		
>4.88	2.28 ± 1.22				
WBC at initial diagnosis					
≤6.27	2.12 ± 1.22	0.028	0.867		
>6.27	2.08 ± 1.00				
NEUT at initial diagnosis					
≤3.50	2.07 ± 1.14	0.061	0.805		
>3.50	2.13 ± 1.10				
MONO at initial diagnosis					
≤0.29	2.13 ± 1.23	0.039	0.844		
>0.29	2.08 ± 1.00				
LY at initial diagnosis					
≤1.95	1.90 ± 1.06	3.182	0.078		
>1.95	2.31 ± 1.13				
PLT/LY at initial diagnosis					
≤117.54	2.38 ± 1.19	6.057	0.016	2.423	0.123
>117.54	1.82 ± 0.96				
NEUT/LY at initial diagnosis					
≤1.74	2.14 ± 1.19	0.118	0.732		
>1.74	2.06 ± 1.04				
LY/MONO at initial diagnosis					
≤6.61	1.93 ± 1.06	2.338	0.130		
>6.61	2.28 ± 1.14				
SII at initial diagnosis					
≤410.69	2.26 ± 1.18	1.776	0.186		
>410.69	1.95 ± 1.03				

**Table 3:** Correlation between CA153 levels and clinicopathological factors at initial diagnosis.

Factor	CA153 value U/mL (mean ± SD)	Univariate analysis		Multivariate analysis	
		*F*	*P*	*F*	*P*
Age					
≤57	11.71 ± 6.01	0.019	0.890		
>57	11.90 ± 7.22				
Menstruation					
Yes	11.52 ± 6.82	0.277	0.600		
No	12.28 ± 6.31				
Comorbidities					
No	11.07 ± 5.57	1.709	0.194		
Yes	12.84 ± 7.70				
BMI					
≤24	11.87 ± 6.17	0.008	0.929		
>24	11.75 ± 6.99				
Tumor size					
≤2 cm	12.20 ± 6.16	0.331	0.567		
>2 cm	11.39 ± 7.09				
Nodal status					
0	11.41 ± 5.83	1.568	0.214		
1-3	13.32 ± 8.37				
≥4	9.60 ± 3.22				
HR status					
Positive	11.87 ± 6.86	0.029	0.865		
Negative	11.59 ± 5.82				
ALT at initial diagnosis					
≤15	11.60 ± 7.07	0.089	0.767		
>15	12.02 ± 6.16				
AST at initial diagnosis					
≤20	11.21 ± 7.06	0.819	0.368		
>20	12.48 ± 6.06				
rGT at initial diagnosis					
≤16	11.20 ± 4.86	1.156	0.285		
>16	12.52 ± 7.86				
Triglycerides at initial diagnosis					
≤1.09	11.58 ± 5.55	0.107	0.744		
>1.09	12.03 ± 7.57				
Cholesterol at initial diagnosis					
≤4.62	10.97 ± 5.55	1.430	0.235		
>4.62	12.64 ± 7.49				
Fasting blood glucose at initial diagnosis					
≤4.88	11.61 ± 6.16	0.080	0.778		
>4.88	12.07 ± 7.10				
WBC					
≤6.27	10.83 ± 5.17	1.962	0.165		
>6.27	12.77 ± 7.72				
NEUT at initial diagnosis					
≤3.50	10.92 ± 5.33	1.575	0.213		
>3.50	12.68 ± 7.63				
MONO at initial					
≤0.29	11.32 ± 6.53	0.459	0.500		
>0.29	12.27 ± 6.71				
LY at initial diagnosis					
≤1.95	11.87 ± 7.52	0.008	0.928		
>1.95	11.74 ± 5.63				
PLT/LY at initial diagnosis					
≤117.54	11.73 ± 5.64	0.013	0.910		
>117.54	11.88 ± 7.51				
NEUT/LY at initial diagnosis					
≤1.74	10.91 ± 5.22	1.602	0.209		
>1.74	12.66 ± 7.66				
LY/MONO at initial diagnosis					
≤6.61	11.37 ± 5.83	0.381	0.538		
>6.61	12.24 ± 7.34				
SII at initial diagnosis					
≤410.69	10.24 ± 5.14	5.049	0.027	5.049	0.027
>410.69	13.30 ± 7.51				

**Table 4:** Effects of postoperative adjuvant chemotherapy on CEA and CA153.

	CEA ng/mL (mean ± SD)	*F*	*P*	CA153 U/mL (mean ± SD)	*F*	*P*
At initial diagnosis (*n* = 90)	2.10 ± 1.11^∗^	8.732	0.000	11.80 ± 6.60^∗^	22.878	0.000
Before chemotherapy (*n* = 90)	1.89 ± 0.82^∗^			12.01 ± 6.22^∗^		
End of chemotherapy (*n* = 90)	2.68 ± 1.34			18.51 ± 8.50		
After 3 months (*n* = 83)	2.45 ± 1.19			10.87 ± 5.96^∗^		

^∗^ *P* < 0.01 compared to that at the end of chemotherapy.

**Table 5:** Analysis of influencing factors of CEA change magnitude during chemotherapy.

Factor	Change magnitude of CEA	Univariate analysis		Multivariate analysis	
		*F*	*P*	*F*	*P*
Age					
≤57	0.42 ± 0.48	0.353	0.554		
>57	0.48 ± 0.49				
Menstruation					
Yes	0.49 ± 0.53	1.229	0.271		
No	0.38 ± 0.39				
Comorbidities					
No	0.39 ± 0.48	2.061	0.155		
Yes	0.53 ± 0.48				
BMI					
≤24	0.47 ± 0.53	0.136	0.713		
>24	0.43 ± 0.44				
Tumor size					
≤2 cm	0.37 ± 0.45				
>2 cm	0.54 ± 0.50				
Nodal status					
0	0.36 ± 0.43	7.560	0.001	15.117	0.000
1-3	0.40 ± 0.45				
≥ 4	0.92 ± 0.52				
HR status					
Positive	0.37 ± 0.43	8.079	0.006	14.834	0.000
Negative	0.70 ± 0.55				
ALT change magnitude					
≤0.13	0.47 ± 0.48	0.294	0.589		
>0.13	0.42 ± 0.48				
AST change magnitude					
≤0.05	0.41 ± 0.46	0.632	0.429		
0.05	0.49 ± 0.50				
rGT change magnitude					
≤0.35	0.31 ± 0.40	8.974	0.004	8.559	0.004
>0.35	0.60 ± 0.51				
Triglyceride change magnitude					
≤0.18	0.44 ± 0.47	0.043	0.835		
>0.18	0.46 ± 0.50				
Cholesterol change magnitude					
≤0.077	0.44 ± 0.56	0.065	0.799		
>0.077	0.46 ± 0.40				
Fasting blood glucose change magnitude					
≤0.05	0.40 ± 0.46	1.113	0.294		
>0.05	0.50 ± 0.50				
Initial WBC					
≤6.27	0.46 ± 0.47	0.045	0.833		
>6.27	0.44 ± 0.50				
Initial NEUT					
≤3.50	0.50 ± 0.47	0.855	0.358		
>3.50	0.40 ± 0.49				
Initial MONO					
≤0.29	0.42 ± 0.43	0.298	0.586		
>0.29	0.48 ± 0.53				
Initial LY					
≤1.95	0.38 ± 0.43	2.010	0.160		
>1.95	0.52 ± 0.53				
Initial PLT/LY					
≤117.54	0.43 ± 0.42	0.203	0.654		
>117.54	0.47 ± 0.54				
Initial NEUT/LY					
≤1.74	0.56 ± 0.50	4.900	0.029	7.078	0.009
>1.74	0.34 ± 0.44				
Initial LY/MONO					
≤6.61	0.45 ± 0.48	0.000	0.982		
>6.61	0.45 ± 0.49				
Initial SII					
≤410.69	0.50 ± 0.46	1.007	0.318		
>410.69	0.40 ± 0.50				

**Table 6:** Analysis of influencing factors of CA153 change magnitude during chemotherapy.

Factor	Change magnitude of CA153	Univariate analysis		Multivariate analysis	
		*F*	*P*	*F*	*P*
Age					
≤57	0.52 ± 0.38	4.632	0.034	0.888	0.349
>57	0.71 ± 0.43				
Menstruation					
Yes	0.67 ± 0.43	2.251	0.137		
No	0.53 ± 0.37				
Comorbidities					
No	0.53 ± 0.38	4.851	0.030	3.144	0.080
Yes	0.72 ± 0.43				
BMI					
≤24	0.63 ± 0.42	0.055	0.814		
>24	0.61 ± 0.41				
Tumor size					
≤2 cm	0.59 ± 0.40	0.342	0.560		
>2 cm	0.64 ± 0.42				
Nodal status					
0	0.59 ± 0.39	7.560	0.026	4.479	0.014
1-3	0.54 ± 0.35				
≥4	0.90 ± 0.53				
HR status					
Positive	0.60 ± 0.40	0.307	0.581		
Negative	0.66 ± 0.45				
ALT change magnitude					
≤0.13	0.57 ± 0.41	0.849	0.359		
>0.13	0.65 ± 0.42				
AST change magnitude					
≤0.05	0.59 ± 0.42	0.406	0.526		
>0.05	0.64 ± 0.40				
rGT change magnitude					
≤0.35	0.55 ± 0.37	2.402	0.125		
>0.35	0.68 ± 0.44				
Triglyceride change magnitude					
≤0.18	0.59 ± 0.44	0.416	0.520		
>0.18	0.64 ± 0.39				
Cholesterol change magnitude					
≤0.077	0.63 ± 0.43	0.187	0.667		
>0.077	0.60 ± 0.40				
Fasting blood glucose change magnitude					
≤0.05	0.56 ± 0.32	1.337	0.251		
>0.05	0.66 ± 0.48				
Initial WBC					
≤6.27	0.75 ± 0.45	10.571	0.002	2.910	0.092
>6.27	0.48 ± 0.32				
Initial NEUT					
≤3.50	0.73 ± 0.43	7.748	0.007	0.657	0.420
>3.50	0.50 ± 0.36				
Initial MONO					
≤0.29	0.42 ± 0.43	0.298	0.586		
>0.29	0.48 ± 0.53				
Initial LY					
≤1.95	0.65 ± 0.46	0.528	0.469		
>1.95	0.59 ± 0.36				
Initial PLT/LY					
≤117.54	0.64 ± 0.43	0.353	0.554		
>117.54	0.59 ± 0.40				
Initial NEUT/LY					
≤1.74	0.67 ± 0.42	1.457	0.231		
>1.74	0.57 ± 0.40				
Initial LY/MONO					
≤6.61	0.64 ± 0.37	0.230	0.633		
>6.61	0.60 ± 0.45				
Initial SII					
≤410.69	0.69 ± 0.44	2.989	0.087		
>410.69	0.54 ± 0.37				

## Data Availability

The raw data supporting the conclusions of this article will be available from the corresponding author on reasonable request.
